# Supplementary material for: Study protocol for the development of a real-time interface showing the availability of breast and cervical cancer services in Ghana
Source: PLoS One. 2024 Oct 17;19(10):e0312150. doi: 10.1371/journal.pone.0312150 (PMC11486384; doi:10.1371/journal.pone.0312150)
Supplement: S1 Appendix — (DOCX) [file pone.0312150.s001.docx]

**S1 APPENDIX I**

# RTIF PROJECT SUMMARY FOR STAKEHOLDER PARTICIPANTS

# Significance (Background)

The management of cancer diseases is a growing public health challenge as incidence rises and outcomes show little improvement. The 5-year survival for breast cancer in Ghana has been estimated up to 39%, whereas in HICs such as the United States and Canada it is nearly 90%.

The striking disparity in patient survival rates is attributed to numerous factors including socioeconomic, cultural, and geographic limitations combined with later stages at diagnosis and more aggressive cancer subtypes, with limited navigability for patients across the healthcare system and limited access to appropriate services for broad sections of the population.

At present time, no known nationally integrated data system exists which may audit hospital capabilities for their self-reference, and comparison and to align these with Ghana’s national priorities and to international standards such as NCCN. While independent verification of these capabilities is possible, such efforts are time-intensive and expensive to maintain. A one-time cross-sectional, hospital-based survey of this scale was performed by the University of Utah/Ensign Global College in collaboration with Ghana Health Services and HeFRA from November 16, 2020 to October 6, 2021.

The survey assessed breast and cervical cancer care capacity at hospitals in Ghana, with all health facilities in Ghana with a hospital designation being approached for participation in the study. The survey aimed to comprehensively describe all hospital-based services available for breast and cervical cancer care in Ghana. The purpose of this study was to determine the existing hospital-based services and their geographic availability nationwide in Ghana, and to identify areas that could benefit most from the targeted expansion of services.

The proposed work seeks to build a real-time interface to explore the possibility that regular, annualized data collection on the location and availability of cancer-focused health services could serve the public good and anchor data products for governmental agencies, private-sector and patient-centric consumption.

The research will assess the overall possibility of developing minimally viable data systems infrastructure focused on facility-level cancer services by considering essential dimensions of precedent, needs, feasibility, and acceptability.

# Study goal and objectives

## Goal

To develop a real-time interface (RTIF) showing the availability of breast and cervical cancer care services at hospital facilities in Ghana.

## Objectives

1. To perform a contextual analysis of the RTIF in the Ghanaian setting
2. To conduct a needs assessment for the RTIF in the Ghanaian setting
3. To execute a feasibility assessment of the RTIF in the Ghanaian setting
4. To create a prototype real-time interface website
5. To evaluate the technical functionality of the RTIF, and further scale up nationwide

# Expected Outcomes of the Study

To date, there has not been such a study leading to this particular significant interface development in Ghana and even in the sub-Saharan African region. The project applies implementation science approaches with the impact and the potential to be far-reaching. A landscape will emerge of “hospital status” health facilities that offer screening, diagnosis, treatment and advocacy for breast and cervical cancer care services for the entirety of Ghana, which will provide the clearest representation to date in acceptable real-time for areas of adequate services as well as those of need. This information can be a valuable guide for both lawmakers and humanitarians regarding where and how to focus future efforts. It will also serve as a reference to patients within and outside Ghana who may be considering traveling for healthcare. It is expected to generate good data for strategic and directional insight into the advancement of cancer control by pharmaceutical and medical technology companies. It will also serve as an aid tool for the regulation and standardization of cancer treatment and referral pathways in Ghana. It is also expected to serve as a model for the improvement of cancer care services in Africa and possibly extend beyond breast and cervical cancer services (the leading cause of cancer-related deaths) to cancer services and other health services in general.
