# Supplementary material for: Study protocol for the development of a real-time interface showing the availability of breast and cervical cancer services in Ghana
Source: PLoS One. 2024 Oct 17;19(10):e0312150. doi: 10.1371/journal.pone.0312150 (PMC11486384; doi:10.1371/journal.pone.0312150)
Supplement: S2 Appendix — (DOCX) [file pone.0312150.s002.docx]

**S2 APPENDIX II**

**KEY INFORMANT INTERVIEW GUIDE**

**(Patients & Survivors)**

**Demographics:**

Can you tell me about yourself?

1. Gender:
2. Age:
3. Highest Education level:
4. What is your profession?
5. Which institution are you currently working with:
6. What is your current position/role:
7. What does this role entail?
8. How many years of work experience do you have:

How long have you had (did you have) this condition?

***Before proceeding to the interview questions:***

1. *Confirm the participant's understanding of the RTIF (Breast and Cervical Cancer Dashboard) Project summary. Present a short summary of what the RTIF project is about before proceeding with the interview if the participant lacks an in-depth understanding of the project.*

**Objective 1: To perform a contextual analysis of the Real-Time Interface (RTIF) in the Ghanaian setting**

*(Intervention Characteristics)*

Evidence Strength & Quality

1. What kind of information or evidence are you aware of that shows whether or not the RTIF (Breast and Cervical Cancer Dashboard) will work in our setting?

o What evidence have you heard about from your own research? Practice guidelines? Published literature? Co-workers? Other patients or survivors? Other settings?

o How does this knowledge affect your perception of the Dashboard?

Relative Advantage

1. How does the Breast and Cervical Cancer Dashboard compare to other similar existing programs in your setting?

o What advantages will the Dashboard have compared to existing health services interfaces or programs?

o What disadvantages will the Dashboard have compared to existing health services interfaces or programs?

2. How does the RTIF (or Dashboard) compare to other alternatives that may have been considered or that you know about at other places/settings within or outside Ghana?

o What advantages will the Dashboard have compared to these other programs?

o What disadvantages will the Dashboard have compared to these other programs?

3. In your opinion, is there another way of making breast/cervical cancer services more accessible?

- Can you describe this alternative?
- Why would people prefer the alternative?

**Objective 2: To conduct a needs assessment for the RTIF in the Ghanaian setting**

Can you talk about the kinds of breast/cervical cancer healthcare services you are currently using (used)?

How did you find out about these services?

- Family sources? Doctor recommendations or referrals? Internet?
- How easy was it to navigate around these services?

*(Outer Setting)*

Patient * Needs

- 1. 1. How well do you think the Dashboard will meet patient needs in Ghana?
- In what ways will the Dashboard meet their needs? E.g. improved access to services? Reduced wait times? Help with self-management? Reduced number of travels for patients and expenses?
  - 1. 2. How do you think patients in Ghana will respond to the Dashboard? And why do you think so?
  1. 3. What barriers will patients in Ghana face using the Dashboard?
  2. 4. What can be done about it?

**Objective 3: To execute a feasibility assessment of the RTIF in the Ghanaian setting.**

***Technical feasibility***

*(Intervention characteristics)*

Design Quality & Packaging

1. What materials (educational, software etc.) do you perceive are needed, for promoting and building of the Dashboard?
2. What materials (educational, software etc.) do you perceive are needed for building the Dashboard?

o Perception on quality of the materials? Why?

o Perception on the packaging of the materials? Why?

- 1. 3. What support, such as online resources, marketing materials, or a toolkit, would you recommend to help with the implementation and use of the Dashboard?

o How can these be accessed?

Ending of Interview

- 1. *Do you have anything to add?*
  2. *Do you have any questions about the dashboard that were not discussed?*

*Thank you for your wonderful insights and for making time for this interview. We will keep you informed of the study's outcome.*
