# Supplementary material for: Study protocol for the development of a real-time interface showing the availability of breast and cervical cancer services in Ghana
Source: PLoS One. 2024 Oct 17;19(10):e0312150. doi: 10.1371/journal.pone.0312150 (PMC11486384; doi:10.1371/journal.pone.0312150)
Supplement: S3 Appendix — (DOCX) [file pone.0312150.s003.docx]

**S3 APPENDIX III**

**KEY INFORMANT INTERVIEW GUIDE**

**(Policymakers & Implementers; Healthcare Managers & Practitioners; NGOs & Research Organizations)**

**Demographics:**

**Can you please tell us about yourself?**

1. Gender:
2. Age:
3. What are your educational qualifications?
4. What is your profession?
5. Which institution are you currently working with:
6. Which Department:
7. What is your current position/role:
8. What does this role entail?
9. How many years of work experience do you have:
10. Do you currently serve on any committees or boards related to cancers (locally and internationally)?

Which ones and in what capacity?

1. Which committees have you served on that are related to cancers (population health) in the past years (locally and internationally)?

Which ones and in what capacity?

***Before proceeding to the interview questions:***

1. *Confirm the participant's understanding of the RTIF (Breast and Cervical Cancer Dashboard) Project summary. Present a short summary of what the RTIF project is about before proceeding with the interview if the participant lacks an in-depth understanding of the project.*

**Objective 1: To perform a contextual analysis of the Real-Time Interface (RTIF) in the Ghanaian setting**

*(Intervention Characteristics)*

Evidence Strength & Quality

1. Do you think the RTIF (Breast and Cervical Cancer Dashboard) will work in our Ghanaian setting? Why do you think it will work/why do you think it will not work?

o What evidence do you have to support it? Practice guidelines? Published literature? Co-workers? Patients? Other settings?

o How does this evidence influence your perception your perception of the Dashboard?

Relative Advantage

1. Do you know of any existing Dashboards in the health sector? [e.g. National Health Insurance Scheme (*NHIS); Lightwave Health Information Management System (LHIMS); Ghana Integrated Logistics Management Information System (GhILMIS); District Health Information Management System (DHIMS)*]
2. How does the Breast and Cervical Cancer Dashboard compare to other similar existing programs in your setting?

o What advantages will the Dashboard have compared to existing health services interfaces or programs?

o What disadvantages will the Dashboard have compared to existing health services interfaces or programs?

1. How does the RTIF (Dashboard) compare to other alternatives that may have been considered or that you know about at other places/settings within or outside Ghana?

o What advantages will the Dashboard have compared to these other programs?

o What disadvantages will the Dashboard have compared to these other programs?

4. In your opinion is there another way of making breast/cervical cancer services more accessible?

- Can you describe this alternative?
- Why would people prefer the alternative?

**Objective 2: To conduct a needs assessment for the RTIF in the Ghanaian setting**

*(Outer Setting)*

- 1. Deliverer (Organizational) and Patient * Needs

1. How well do you think the Dashboard will meet the priority programs and activities of organizations (organizational goals/objectives) in Ghana? Particularly your organization?

- In what ways will the Dashboard meet their needs? E.g. improved patient access to services? Reduced wait times? Help with patient management? Reduced number of travels for patients and expenses?

1. a. How do you think individuals (including patients) in Ghana will respond to the Dashboard? And why do you think so?

b. How do you think organizational stakeholders in Ghana will respond to the Dashboard? And why do you think so?

1. What barriers will individuals (including patients) and stakeholders in Ghana face using the Dashboard?

*(Lack of Internet access, Illiteracy, Poverty)*

3b. What barriers will organizational stakeholders in Ghana face using the Dashboard?

*(Work overload, Bureaucracy, Political will)*

1. What can be done about it?

**Objective 3: To execute a feasibility assessment of the RTIF in the Ghanaian setting**

***Technical feasibility***

*(Intervention characteristics)*

Design Quality & Packaging

1. What materials (educational, software etc.) do you perceive are needed, for promoting the Dashboard?
2. What materials (educational, software etc.) do you perceive are needed for building the Dashboard?

o Perception on quality of the materials? Why?

o Perception on the packaging of the materials? Why?

- 1. 3. What support, such as online resources, marketing materials, or a toolkit, would you recommend to help with the implementation and use of the Dashboard?
  2. o How can these be accessed?
  3. 4. How will existing electronic health systems/databases influence the implementation of the Dashboard in the Ghanaian setting?

o Name and explain how each will affect the Dashboard (positively and negatively)

- 1. 5. How can the Dashboard synchronize with these existing electronic health systems?

***Economic feasibility***

Resources *(inner setting)* and Costs *(intervention characteristics)*

1. What are the perceived resources needed to implement the Dashboard in Ghana and in your organization or facility?

*(Resources may include operating and capital funding, dedicated personnel time (e.g. have new staff been hired, or is implementation a collateral duty), space, equipment, and/or information technology)*

2. What costs should be considered to implement the Dashboard?

o Direct and indirect costs? Short and long term?

***Operational feasibility***

(Outer setting)

External Policies & Incentives

*[External policies on information systems and management (such as DHIMS etc.) and regulations surrounding its management. Local and international indicators (measures) or guidelines for measuring health services performance in Ghana]*

- 1. 1. What kind of local, state, national, or international performance measures, policies, regulations, or guidelines do you perceive will influence the implementation of the Dashboard?

o Given the mentioned, how will the Dashboard affect your organization as well as other organization's ability to meet them (i.e. these measures, policies, regulations, or guidelines)?

- 1. o Will this be positive and/or negative?

*(Inner setting)*

Structural Characteristics/culture

*(This construct can be used to capture any relevant information regarding the social architecture, culture, electronic systems or physical layout of the organization)*

- 1. 1. How will the structure (social architecture, electronic systems or physical layout) of your organization as well as other organizations’ affect the implementation of the Dashboard?
  2. o How will the infrastructure facilitate/hinder implementation of the Dashboard?
  3. o How can such structural challenges be addressed?
  4. o Changes in formal policies? Changes in information systems or electronic records systems? Other?
  5. o What kind of approvals will be needed? Who will need to be involved? [e.g. Public Health Unit, Policy Planning Monitoring & Evaluation (PPME) unit)
  6. o Can you describe the process that will be needed to make these changes?

Implementation Climate

- 1. 1. What could be the general level of receptivity in your organization to the implementation of the Dashboard?
  2. o Why?

Ending of Interview

- 1. *Do you have anything to add?*
  2. *Do you have any questions about the dashboard that were not discussed?*

*Thank you for your wonderful insights and for making time for this interview. We will keep you informed of the study's outcome.*
