# Supplementary material for: Study protocol for the development of a real-time interface showing the availability of breast and cervical cancer services in Ghana
Source: PLoS One. 2024 Oct 17;19(10):e0312150. doi: 10.1371/journal.pone.0312150 (PMC11486384; doi:10.1371/journal.pone.0312150)
Supplement: S4 Appendix — (DOCX) [file pone.0312150.s004.docx]

**S4 APPENDIX IV**

**Objective 5: To evaluate the technical functionality of the RTIF and further scale up nationwide.**

***** *Selected stakeholders (organizational informants and cancer patients/survivors)*

Participant number: _____

**System Usability Scale (SUS)**

This is a standard questionnaire that measures the overall usability of a system. Please select the answer that best expresses how you feel about each statement after using the website (the RTIF).

|  | Strongly Disagree | Somewhat Disagree | Neutral | Somewhat Agree | Strongly Agree |
| --- | --- | --- | --- | --- | --- |
| 1. I think I would like to use this tool frequently. |  |  |  |  |  |
| 1. I found the tool unnecessarily complex. |  |  |  |  |  |
| 1. I thought the tool was easy to use. |  |  |  |  |  |
| 1. I think that I would need the support of a technical person to be able to use this system. |  |  |  |  |  |
| 1. I found the various functions in this tool were well integrated. |  |  |  |  |  |
| 1. I thought there was too much inconsistency in this tool. |  |  |  |  |  |
| 1. I would imagine that most people would learn to use this tool very quickly. |  |  |  |  |  |
| 1. I found the tool very cumbersome to use. |  |  |  |  |  |
| 1. I felt very confident using the tool. |  |  |  |  |  |
| 1. I needed to learn a lot of things before I could get going with this tool. |  |  |  |  |  |

How likely are you to recommend this website to others? (please circle your answer)

Not at all likely 0 1 2 3 4 5 6 7 8 9 10 Extremely likely
