# Supplementary material for: Study protocol for the development of a real-time interface showing the availability of breast and cervical cancer services in Ghana
Source: PLoS One. 2024 Oct 17;19(10):e0312150. doi: 10.1371/journal.pone.0312150 (PMC11486384; doi:10.1371/journal.pone.0312150)
Supplement: Attachment — Submitted filename: Response to Reviewers.pdf [file pone.0312150.s005.pdf]

POINT BY POINT RESPONSE TO COMMENTS

JOURNAL REQUIREMENTS ADDRESSED:

1. **Please ensure that your manuscript meets PLOS ONE's style requirements, including those for file naming. The PLOS ONE style templates can be found at https://journals.plos.org/plosone/s/file?id=wjVg/PLOSOne_formatting_sample_main_body.pdf and https://journals.plos.org/plosone/s/file?id=ba62/PLOSOne_formatting_sample_title_authors_affiliations.pdf**

   The manuscript has been examined to ensure it meets the PLOS ONE style templates shared, please. Particularly the headings have been revised.

2. **Thank you for stating the following in your Competing Interests section: "The authors declare that this research could evolve into a self-sustaining project by exploring and leveraging viable start-up opportunities. They affirm that this competing interest has not influenced the design, execution, or interpretation of the study."**
   Please complete your Competing Interests on the online submission form to state any Competing Interests. If you have no competing interests, please state ""The authors have declared that no competing interests exist."", as detailed online in our guide for authors at http://journals.plos.org/plosone/s/submit-now
   This information should be included in your cover letter; we will change the online submission form on your behalf.

   This has been addressed. Also, this competing interest is placed in the cover response letter.

3. **In the online submission form, you indicated that no datasets have been generated or analysed during the project. All relevant data from this study will be made available upon study completion and upon reasonable request. The data-sharing and ownership within the GHS approved ethics study protocol requires the data to be kept under the supervision of the research team at Ensign Global College/Center for Global Surgery and de-identified data made available upon request for researchers who meet the criteria for access to confidential data. For access to the data, requests may be sent to the institution at globalsurgery@hsc.utah.edu.**
   All PLOS journals now require all data underlying the findings described in their manuscript to be freely available to other researchers, either 1. In a public repository, 2. Within the manuscript itself, or 3. Uploaded as supplementary information.
   This policy applies to all data except where public deposition would breach compliance with the protocol approved by your research ethics board. If your data cannot be made publicly available for ethical or legal reasons (e.g., public availability would compromise patient

privacy), please explain your reasons on resubmission and your exemption request will be escalated for approval.

This statement has been revised to,

"*This is a protocol manuscript in which the study has not yet completed data collection at the time of submission and not yet generated results or findings from data analysis. No data or findings is thus reported within this protocol manuscript. Any derivative manuscripts from the project shall include all data underlying the findings within the manuscripts*."
And this has been iterated in the cover letter to provide clarity.

4. **Your ethics statement should only appear in the Methods section of your manuscript. If your ethics statement is written in any section besides the Methods, please delete it from any other section.**

   The ethics statement has now been merged into the Materials and Methods section of the manuscript.

5. **Please include captions for your Supporting Information files at the end of your manuscript, and update any in-text citations to match accordingly. Please see our Supporting Information guidelines for more information: http://journals.plos.org/plosone/s/supporting-information.**

   Revisions have been applied to the captions of the Supporting Information files at the end of the manuscript and the in-text citations have also been revised to match accordingly.

<u>ADDITIONAL EDITOR COMMENTS:</u>

Thank you for submitting your manuscript to Plos one.

I have completed my evaluation of your manuscript. The reviewers recommend reconsideration of your manuscript following major revision. I invite you to resubmit your manuscript after addressing the comments below.

1. **The authors have mentioned that the information obtained from the study shall be a valuable guide for both lawmakers and humanitarians regarding where and how to focus future efforts. The authors are encouraged to briefly describe about the present policy status and its shortfall. As well as the end results of this study would make improvement for the public in framing the future of cancer patients in Ghana. Thanks**

A description of related policies and shortfalls with relevant citations have been added to page 21; line 456 of the track-changed manuscript to address this concern:

"*In the year 2011, the national strategy for cancer control in Ghana (2012-2016) was created and it detailed out an implementation plan on how to integrate cancer care services into all levels of the healthcare system. Breast and cervical cancers were top amongst the list of seven identified priority cancers [21]. The strategy document is yet to be updated. It, however, underscores the importance of having a tool such as the RTIF to guide policy-making with up-to-date data for progressive evaluation. Again, the national policy on non-communicable diseases (NCDs) , in relation to cancers, currently aims at strengthening existing structures for the prevention and control of cancers within the country through the promotion of routine screening, increasing financial access to quality care for cancer and the strengthening of cancer surveillance[22]. This policy emphasizes joint collaboration between NGOs (humanitarians) and the government (lawmakers) towards the achievement of the agenda. Data outputs from the RTIF shall provide helpful information for the aforementioned for decision and intervention purposes. In this regard, the RTIF will thus help improve the management framework of future cancer patients in Ghana*."

REVIEWER #1:

The current manuscript emphasizes the study protocol and its availability of breast and cervical cancer services in Ghana. Compared with existing reports, the topic of the work was very interesting. There are some suggestions for improvement about this manuscript as follows,

1. **Incidence of breast and cervical cancer a worldwide statistics and recent data can be cited.**

This has been included in the introduction section of the track change manuscript on page 5, line 89 with relevant citations:

"*Each year, about 2.1 million women worldwide are diagnosed with breast cancer, making it the leading cause of cancer-related deaths among women globally. In 2018, an estimated 627,000 women died from breast cancer, with the majority of these deaths occurring in sub-Saharan Africa (SSA) [1]. For cervical cancer, according to the 2020 GLOBOCAN report, there were 604,000 new cases and 342,000 deaths related to the disease worldwide. Approximately 90% of these deaths occurred in low- and middle-income countries [2].*"

2. **The detailed study selection process and its framework can be included.**

   The study is a primary qualitative survey. Respondents will be selected purposively to respond to a modified interview guide derived from the updated Consolidated Framework for Implementation Research (CFIR) and which align with the study's specific objectives[14,17]. This study is not a systematic or scoping review, and hence a study selection process and framework to achieve the set-out objectives is not required or typically included.

   We have described this in **the conceptual framework subsection** within the materials and methods section of the track changed manuscript on page 8 with the associated citation reference leading to the framework details. It is further expatiated in the data collection tools for objectives 1,2 &3 subsection on pages 10 and 11, please. Thank you.

3. **History background related to this condition is not included in the questionnaire.**

   We appreciate this comment. The related history background to the research project was initially omitted but has now been included in the appendix as S1 Appendix I. The related interview guides for the stakeholder participants have thus been renamed S2 Appendix II and S3 Appendix III accordingly. S3 is now renamed as S4 Appendix IV. Thank you.

4. **Pictorial representation of study design, data analysis and management plan can be included for the better representation.**

   We appreciate this insightful comment. The authors attempted to capture this in pictorial representation as suggested; however, the nature of the research is such that it cannot be comprehensively expressed as such. We found it simpler to omit such a presentation but anticipate the creation of such a diagram after the study's completion to account for the study findings in relation to the design, data analysis, outcomes, and their interconnectedness. Thank you.

5. **What is the sample size of the study and how it can be categorized? Also age wise severity on breast and cervical cancer.**

   The study involves qualitative interviews of stakeholders in the cancer care continuum within Ghana. Under the **sampling and data collection subsection of the method section**, on page 10 of the track changed manuscript, line 208, we had stated:

   "The enrolment of participants will be categorized broadly as follows policy makers and implementers; healthcare managers and practitioners; NGOs and research organizations; breast/cervical cancer patients and survivors. A fair representation of these stakeholders shall guide the selection of the respondents. Anticipated respondents will be from the Ministry of

Health, Ghana Health Services, Health Facilities Regulatory Agency (HeFRA), Ghana Breast Society etc. It is anticipated that saturation point shall be attained after conducting between 9 -17 in-depth interviews, however the target number of individual in-depth interviews shall be approximately 35-40 (4)*".*

The last sentence within this paragraph has been reviewed to, "*It is anticipated that saturation point shall be attained after conducting between 9 -17 in-depth interviews. However, the target number of individual in-depth interviews shall be approximately 35-40 due to expected heterogeneity in the sample* (4)."

The individual in-depth qualitative interviews of the mentioned stakeholder groups shall proceed until saturation is reached, as described in the methods.
For this study and its nature, age-wise severity of breast and cervical cancers is unlikely to be of relevance however, it will be coded during data extraction if the topic emerges during analysis.

We hope this answers the concern raised, please.

6. **Add service components which implies the services available and a hand out which has illustrative pictures with explanations in the language of their preference.**

The selection of participants for this study shall be purposive. The stakeholder participants (policy makers and implementers; healthcare managers and practitioners; NGOs and research organizations; breast/cervical cancer patients and survivors) will be selected due to their knowledge and experience in relation to the management of these diseases. The demography of these respondents is such that they understand the types and nature of services around the study. All respondents are expected to be English language literate.

Including a handout for this purpose will thus be redundant, please.

7. **Analyse the advantages, disadvantages, and expenses of various follow-up procedures for cervical and breast cancer patients who have finished their primary treatment.**

The objectives of this research study are not to understand the entirety of the cancer care continuum but are delineated explicitly as:

Objective 1: To perform a contextual analysis of the RTIF in the Ghanaian setting.
Objective 2: To conduct a needs assessment for the RTIF in the Ghanaian setting.
Objective 3: To execute a feasibility assessment of the RTIF in the Ghanaian setting.
Objective 4: To create a prototype real-time interface website.
Objective 5: To evaluate the technical functionality of the RTIF, and further scale up nationwide

The advantages, disadvantages, and expenses of various follow-up procedures for cervical and breast cancer patients who have finished their primary treatment will, however be coded

during data extraction if the topics emerge under the study objectives during data analysis for discussion and address within the parameters of the study, please.

8. **References in manuscript and the formatting can be arranged as per author guidelines of the journal.**

   The references in the manuscript and the formatting have been proof-checked and identified inconsistencies have been addressed accordingly.

REVIEWER #2 ADDRESSED:

The paper outlines a study protocol for developing a real-time interface (RTIF) that shows the availability of breast and cervical cancer services in Ghana. The primary goal of this work is to improve the accessibility and navigability of cancer care services in Ghana, addressing the low 5-year survival rates for breast and cervical cancers compared to developed countries. The project involves multiple phases, including contextual analysis, needs assessment, feasibility assessment, prototype development, and usability testing, guided by the updated Consolidated Framework for Implementation Research (CFIR). There was clarity and organization in the study and each section is well-organized and providing clear, detailed information relevant to the study's objectives and methods. The introduction and background sections thoroughly explain the context, significance, and rationale for the study, setting a solid foundation for understanding the research goals. The methodology is meticulously detailed, outlining each step of the research process and ensuring replicability. Ethical considerations are comprehensively addressed, demonstrating the study's adherence to ethical research standards. Additionally, the use of the Consolidated Framework for Implementation Research (CFIR) is well-integrated into the study design, providing a robust framework for guiding and evaluating the research. However, there are a few clarifications that need to be addressed by the authors before proceeding this paper to the next stage of the publication.

1. The paper mentions the low 5-year survival rates for breast and cervical cancer in Ghana compared to developed countries. What specific gaps in the current healthcare system does this study aim to address, and how will the real-time interface (RTIF) improve patient outcomes?

Thank you for this insightful comment. This concern is similar to that raised in the additional editor’s comments, and the authors have addressed this on page 21, line 456 of the track changed manuscript:

"In the year 2011, the national strategy for cancer control in Ghana (2012-2016) was created and it detailed out an implementation plan on how to integrate cancer care services into all levels of the healthcare system. Breast and cervical cancers were top amongst the list of seven identified priority cancers [21]. The strategy document is yet to be updated. It, however, underscores the importance of having a tool such as the RTIF to guide policy-making with up-to-date data for progressive evaluation. Again, the national policy on non-communicable diseases (NCDs), in relation to cancers, currently aims at strengthening existing structures for the prevention and control of cancers within the country through the promotion of routine screening, increasing financial access to quality care for cancer and the strengthening of cancer surveillance [22]. This policy emphasizes joint collaboration between NGOs (humanitarians) and the government (lawmakers) towards the achievement of the agenda. Data outputs from the RTIF shall provide helpful information for the aforementioned for decision and intervention purposes. In this regard, the RTIF will thus help improve the management framework of future cancer patients in Ghana."

2. The study will use purposive sampling and snowballing techniques for stakeholder interviews. How will the researchers ensure a representative sample of stakeholders, and what criteria will be used to identify key opinion leaders in the breast and cervical cancer care community?

The study involves qualitative interviews of stakeholders in the cancer care continuum within Ghana and they shall be enrolled using purposive sampling and snowballing technique.

Under the sampling and data collection subsection of the methodology, on page 10 of the track changed manuscript we stated:

"The stakeholders and key opinion leaders (KOL) identified shall be purposefully chosen based on function in the Ghanaian health system and relevance to the study objectives.
The enrolment of participants will be categorized broadly as follows policy makers and implementers; healthcare managers and practitioners; NGOs and research organizations; breast/cervical cancer patients and survivors. A fair representation of these stakeholders shall guide the selection of the respondents. Anticipated respondents will be from the Ministry of Health, Ghana Health Services, Health Facilities Regulatory Agency (HeFRA), Ghana Breast Society etc. It is anticipated that saturation point shall be attained after conducting between 9 - 17 in-depth interviews. However, the target number of individual in-depth interviews shall be approximately 35-40 due to expected heterogeneity in the sample (4)."

The inclusion criteria for the identification of stakeholders is being part of the four categorized areas:
policy makers and implementers; healthcare managers and practitioners; NGOs and research organizations; breast/cervical cancer patients and survivors.

The respondents shall be selected based on their function in the Ghanaian health system and relevance to the study objectives as stated in the section.

The authors have included this additional sentence on line 202, of page 10 within the same section to detail the inclusion criteria:

"*These shall include individuals at organizations with significant years of experience working within the healthcare continuum and recognized by peers as having significant years of experience in leadership roles, significant contributions to research or clinical practice, as well as having reputation and/or influence in policy-making or guideline development in Ghana in relation to cancers (breast and cervical). Survivors and patients of these cancers shall also be included*."

To ensure a representative sample of stakeholder responses, approximately 35-40 individual in-depth interviews will be conducted, taking into account the expected heterogeneity within the sample

3. Ethical approval has been obtained from both Ghana Health Service Ethics Review Committee and the University of Utah Institutional Review Board. What specific ethical challenges might arise during this study, and how does the study protocol plan to mitigate them?

There may arise the issue of location (country) of storage of the data generated through the RTIF on healthcare services relating to breast and cervical cancer services.

On page 12, line 257 of the track changed manuscript, the statement,
"*This shall involve a REDCap-based 'database' for prototyping, leveraging an instance at University of Utah and shall be merged with HeFRA online systems*"

has been revised to:

"*This will involve developing a prototype using a REDCap-based database, initially hosted at the University of Utah. The database will later be transferred to Ensign Global College's REDCap system in Ghana and integrated with HeFRA's online platforms*"

On page 20, line 436 of the track changed manuscript, **under the ethical considerations section,** a statement has been inserted to account for this:
"*All data generated through the RTIF shall be stored on a secure server in Ghana based at the Ensign Global College, and administrative rights and access will be given to only authorized persons connected with the study*."

4. The study employs the Consolidated Framework for Implementation Research (CFIR) to guide the development of the RTIF. Can the authors elaborate on how each of the four domains (intervention, outer setting, inner setting, and individual characteristics) will specifically influence the design and implementation of the RTIF?

This comment is well noted and has been addressed by including the following details **under the conceptual framework section** on page 8 and line 167,

"Specifically, exploration of these four domains is expected to influence the design and implementation of the RTIF as follows:

- *i.* ***Intervention Characteristics****: It will ensure the RTIF is adaptable, user-friendly, and compatible with existing health systems to enhance its adoption.*
- *ii.* ***Outer Setting****: It will help address external factors like patient needs and the broader healthcare environment to align the RTIF with national priorities and stakeholder expectations.*
- *iii.* ***Inner Setting****: It shall evaluate organizational culture, readiness, and resource availability to integrate the RTIF into existing workflows effectively.*
- *iv.* ***Individual Characteristics****: It allows to customize training and support based on users' skills and attitudes to ensure they can use the RTIF confidently.*

This aforementioned and described comprehensive approach ensures the RTIF is tailored to the Ghanaian context, promoting successful implementation and sustainability."

5. The study mentions the integration of service availability data into the RTIF. How will the researchers ensure the accuracy and reliability of the data entered by healthcare facilities, and what measures are in place to regularly update this information?

HeFRA is a collaborator in this research project. The following statement has been included within the manuscript on page 13, line 274 in address to the concern raised, please.

"HeFRA is legally mandated to license and monitor all healthcare facilities in Ghana to ensure quality public and private healthcare delivery services. Before any data is displayed on the RTIF, HeFRA will verify its accuracy and reliability through quality checks conducted by its regional officers. Additionally, health facilities are required to report any changes in their service delivery operations and status to HeFRA. To maintain accurate service availability data, HeFRA will regularly and periodically enforce these reporting requirements, ensuring updates are made at defined intervals so as to ensure currency of information."

THANK YOU.
